# Exploration of the Chloride Binding Behavior of Anhydrous Calcium Sulfoaluminate Under Dual Chloride Ingress Modes

**DOI:** 10.3390/ma18214949

**Published:** 2025-10-30

**Authors:** Zirui Cheng, Luyan Ji, Zhen Wang, Linlin Gu, Wenbin Tang

**Affiliations:** 1 School of Safety Science and Engineering, Nanjing University of Science and Technology, Nanjing 210094, China; chengzr@njust.edu.cn (Z.C.);; 2 School of Mechanical Engineering, Nanjing University of Science and Technology, Nanjing 210094, China

**Keywords:** anhydrous calcium sulfoaluminate, chloride binding capacity, internal chloride incorporation, external chloride penetration

## Abstract

This study explored the chloride binding characteristics and mechanisms of sulphoaluminate cement (SAC) by isolating its principal mineral component, anhydrous calcium sulphoaluminate ($C_{4}A_{3}\overset{-}{S}$), as the research object. Chloride ingress was investigated under external penetration and internal incorporation conditions, with gypsum dosage varied at molar ratios of 1:0, 1:1, and 1:2 relative to ${\;C}_{4}A_{3}\overset{-}{S}$. Through chloride binding experiments and hydration product analysis performed by XRD and TG, the following findings were obtained: under external chloride exposure, the binding capacity increased with rising solution concentration and immersion time. External chloride binding was attributed to SO_4_^2−^/Cl^−^ ion exchange in AFm to generate Friedel’s salt and was complemented by physical adsorption of chloride in AH_3_ gel. Under internal chloride incorporation, binding capacity increased progressively with curing age. Internal chloride binding involved the direct participation of Cl^−^ in hydration reactions to form Friedel’s salt in addition to the chemical reaction of AFm and the physical adsorption of AH_3_. Gypsum dosage critically regulates the AFm/AFt ratio, which in turn governs chloride binding efficiency under both external and internal chloride scenarios (e.g., after immersion in 1 mol/L NaCl solution, the bound chloride content for $C_{4}A_{3}\overset{-}{S}$/gypsum ratios of 1:0, 1:1, and 1:2 was 50.94, 27.28, and 13.47 mg/g, respectively).

## 1. Introduction

In recent years, driven by the urgent requirement for sustainable development and marine strategy, the global focus on marine resource exploration and utilization has intensified [1]. As the cornerstone of offshore infrastructure, concrete structures play a vital role in various marine engineering projects. However, the long-term exposure to the marine environment causes concrete structures to be corroded by various corrosive ions in seawater, and chloride-induced corrosion of steel reinforcement remains the most critical durability issue, leading to expansive rust formation, cracking, and structural degradation. Currently, Portland cement (PC)-based concrete dominates the construction industry; it still faces two major limitations in marine environments: high carbon footprint and insufficient chloride resistance. The production of PC accounts for approximately 8% of global CO_2_ emissions [2], which conflicts with stringent environmental targets. Moreover, PC-based concrete exhibits limited resistance to seawater erosion due to its vulnerable microstructure and susceptibility to chemical attacks, exacerbating the chloride-induced degradation in marine environments. These limitations require the application of new cementitious materials with enhanced resistance to chloride ion attack and low carbon emissions to ensure the longevity and sustainability of marine infrastructure.

Meanwhile, sulfoaluminate cement (SAC) has emerged as a promising alternative to Portland cement, particularly in addressing the challenges associated with high carbon emissions and chloride erosion in marine engineering. Since the main mineral component in SAC clinker is ye’elimite, also called anhydrous calcium sulfoaluminate (4CaO·3Al_2_O_3_·SO_3_, $C_{4}A_{3}\bar{S}$) (note that standard cement nomenclature is followed here, whereby C = CaO, S = SiO_2_, A = Al_2_O_3_, $\bar{S}$ = SO_3_, and H = H_2_O), and a small amount of belite (C_2_S), it requires less limestone during the production process, which can significantly reduce CO_2_ emissions [3]. In addition, the calcination temperature of SAC is approximately 150 ℃ lower than that of OPC, and SAC clinker is easier to grind, which can reduce the energy consumption of the SAC production process and indirectly reduce CO_2_ emissions associated with the fuel combustion [4]. In addition to the low carbon characteristics, the hydration of $C_{4}A_{3}\overset{-}{S}$ will form ettringite (AFt), AFm phase, and aluminum hydroxide gel (AH_3_), which endows SAC with good corrosion resistance. The chloride diffusion coefficient of SAC was reported to be much lower than that of OPC, which is due to the dense pore structure formed by its unique hydration product system [5,6]. In addition, long-term exposure tests of SAC concrete in tidal zones have shown that SAC exhibited greater strength stability than that of PC concrete when exposed to drying–wetting cycles in a marine environment [7,8].

The chloride binding capacity of cement-based materials is also an important indicator of the resistance to chloride erosion [9]. Chloride ions invading from the external environment or introduced by raw materials can be partially captured by hydration products as bound chloride ions, reducing the content of free chloride ions inside concrete and thereby delaying the electrochemical corrosion of steel bars [10,11,12]. Numerous studies have investigated the chloride binding behavior of OPC, and it is widely recognized that C-S-H phases dominate this process, primarily through physical adsorption [13,14]. In contrast, AFm phases chemically bind free chloride ions by forming Friedel’s salt [15,16].

With regard to SAC, studies have demonstrated that SAC exhibits a certain chloride binding capacity; chloride can either be chemically bound to form Friedel’s salt or be physically adsorbed by other hydration products [17,18]. Nevertheless, the capacity is relatively lower than that of OPC, potentially resulting in a higher free chloride content in SAC compared to OPC after prolonged exposure to seawater [19,20]. In addition, the calcium sulfate content and the water-to-cement (W/C) ratio have been reported to influence SAC’s chloride binding capacity. Specifically, a higher sulfate content restricts the formation of AFm phases, whereas a higher W/C ratio promotes a higher degree of hydration, thereby yielding more hydration products that can contribute to chloride binding [21,22]. Furthermore, due to the distinct composition of SAC’s hydration products compared to OPC, the chloride binding mechanism also differs; there still lacks a comprehensive investigation of the chloride binding mechanism of SAC. Since commercial SAC products typically consist of a complex assemblage of mineral components, leading to a complex hydration process with multiple products, it makes it difficult to analyze the key reactions and individual mineral contributions to chloride binding. Notably, $C_{4}A_{3}\overset{-}{S}\;$ is the dominant mineral component of SAC and the main source for the hydration products involved in chloride binding. Using pure $C_{4}A_{3}\overset{-}{S}\;$ for research can effectively eliminate the interference of other mineral components and facilitate the revelation of chloride binding mechanisms.

By isolating pure $C_{4}A_{3}\overset{-}{S}\;$ as the research object, the complex hydration system of commercial SAC can be simplified, facilitating a more precise investigation of chloride binding behaviors of SAC. A series of chloride binding experiments were conducted using pure $C_{4}A_{3}\overset{-}{S}\;$ minerals, incorporating both external invading and internal mixing chloride ions. Additionally, the influence of gypsum content on the hydration process and chloride binding behavior was evaluated. X-ray diffraction (XRD) and thermogravimetry (TG/DTG) were used for analyses of the hydration products after chloride binding was complete. This study aims to clarify the binding process and the factors influencing the chloride binding of $C_{4}A_{3}\overset{-}{S}$ as well as the underlying mechanisms for both internal and external chloride ions.

## 2. Materials and Experimental Design

### 2.1. Experimental Materials and Sample Preparation

**Figure 1:**
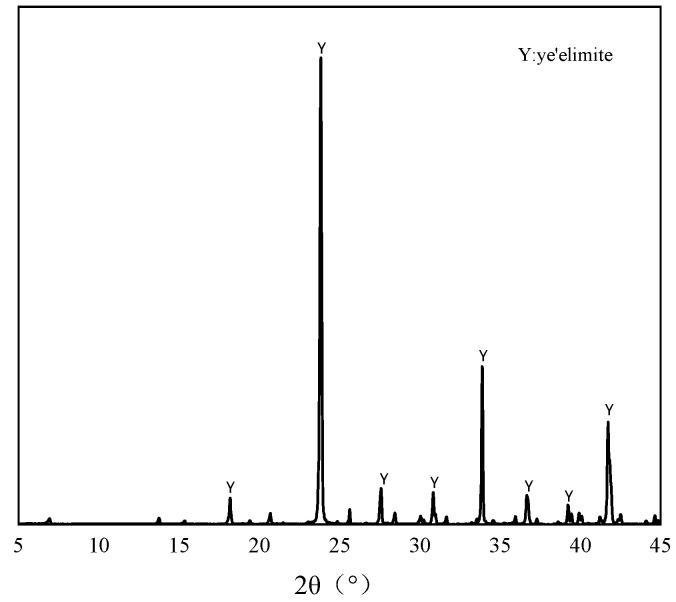
XRD patterns of the synthesized $C_{4}A_{3}\overset{-}{S}$.

The pure $C_{4}A_{3}\overset{-}{S}$ minerals used in this study were synthesized through the following procedure: Analytical-grade CaO, Al_2_O_3_, and CaSO_4_ (provided by Sinopharm Chemical Reagent Co., Ltd., Shanghai, China) were precisely weighed in a 3:3:1 molar ratio and mixed well. The mixture was moistened with deionized water and compacted into cylindrical specimens (φ50 mm × 10 mm) using a hydraulic press. The specimens were subjected to sequential thermal treatment, beginning with 1 h drying at 110 °C, followed by calcination in a high-temperature furnace. The calcination protocol involved heating to 1350 °C at a controlled ramp rate, holding for 2 h to ensure complete solid-state reaction, and subsequently quenching rapidly to ambient temperature [23]. The resulting material was then ground to pass through an 80-μm sieve for storage [24]. The XRD pattern and the chemical compositions of the prepared $C_{4}A_{3}\overset{-}{S}$ are shown in Figure 1 and Table 1. Analytical grade dihydrate gypsum was used to regulate the hydration of the $C_{4}A_{3}\overset{-}{S}$ and NaCl was used as the source of Cl^−^. To ensure the accuracy of the total chloride ion dosage during the experiment, deionized water was applied to prepare the samples.

The hydration process of $C_{4}A_{3}\overset{-}{S}$ is strongly dependent on gypsum content. In the absence of gypsum, $C_{4}A_{3}\overset{-}{S}$ primarily hydrates to form the AFm phase, accompanied by the generation of aluminum hydroxide gel (AH_3_). In contrast, abundant gypsum promotes ettringite (AFt) formation alongside AH_3_, as illustrated in Equations (1) and (2) [25]. Consequently, the $C_{4}A_{3}\overset{-}{S}$-to-gypsum ratio governs the relative proportions of these hydration products, ultimately determining the chloride binding capacity.

(1) $$C_{4}A_{3}\overset{-}{S}+18H\to C_{3}A\cdot C\overset{-}{S}\cdot 12H+2AH_{3}$$

(2) $$C_{4}A_{3}\overset{-}{S}+2C\overset{-}{S}H_{2}+34H\to C_{3}A\cdot 3C\overset{-}{S}\cdot 32H+2AH_{3}$$

In this study, to investigate the chloride binding capacity and underlying mechanism of SAC with varying gypsum dosages, samples were prepared with molar ratios of $C_{4}A_{3}\overset{-}{S}$-to-gypsum of 1:0, 1:1, and 1:2, respectively. A constant water-to-cement ratio of 0.4 was maintained for all mixtures. After accurately weighing the raw materials and mixing them with water in the designated proportions, the mixtures were stirred thoroughly. The mixtures were then poured into molds and vibrated to eliminate air bubbles. After 24 h, the samples were demolded and transferred to a standard curing room (20 ± 2 °C, RH 98%) for curing until the designated testing age. The detailed mix proportion is shown in Table 2.

### 2.2. Chloride Ion Binding Experiment

Chloride ions can invade into concrete through different pathways: (1) permeation from external marine environments via diffusion and capillary action, or (2) direct incorporation during mixing through chloride-containing raw materials (e.g., sea sand and seawater). Consequently, chlorides can be classified into internally admixed or externally penetrated types. This study investigates the chloride binding behavior of $C_{4}A_{3}\overset{-}{S}$ and underlying mechanisms under both invasion modes through comparative experiments.

(1) Externally penetrated chloride ions

To simulate chloride invasion in marine environments, specimens were subjected to external chloride penetration through immersion in NaCl solutions. This experiment evaluated the chloride binding performance of $C_{4}A_{3}\overset{-}{S}$ under varying gypsum dosages, immersion durations (3 h, 6 h, 12 h, 1 day, and 3 days), and chloride ion concentrations (0.2, 0.4, 0.6, 0.8, and 1.0 mol/L). Following the mix proportions specified in Table 2, specimens with 28 days curing age were ground and sieved to a particle size ≤0.2 mm. For each mix, 4 g samples were immersed in 20 mL of NaCl solution, and periodic shaking was applied to enhance solid–liquid interactions. For the analysis of the time-dependent binding behavior, specimens were immersed in a 0.6 mol/L NaCl solution for varying durations. For concentration-dependent studies, samples were immersed in NaCl solution with different chloride concentrations for 28 days. After immersion, the supernatant was collected to measure the chloride concentration using automatic potentiometric titration. The solid residues were rinsed with deionized water, oven-dried at 60 °C for 24 h, ground to a particle size of ≤0.075 mm, and stored in sealed containers for further analysis.

(2) Internally incorporated chloride ions

To simulate the production of concrete using sea sand, NaCl was directly incorporated into the mixture during specimen preparation. This study examined the chloride binding capacity of $C_{4}A_{3}\overset{-}{S}$ under different gypsum dosages, curing ages (3, 7, and 28 days), and internally admixed chloride concentrations (0.02, 0.04, and 0.06 mol). The preparation of samples also followed the mix proportions presented in Table 2, with the specified amounts of chloride introduced during mixing. After the designated curing ages were reached, the hydration was terminated by immersing the samples in anhydrous ethanol, followed by oven drying, grinding, and sieving to a particle size of ≤0.2 mm. Two grams of powdered sample were agitated in 40 g of deionized water for 2 h and then allowed to settle for 24 h. Then the supernatant was also collected to measure chloride ion concentration using automatic potentiometric titration. The residual solids were hermetically sealed for subsequent material characterization.

(3) Methodology for assessing chloride binding capacity

The chloride binding capacity was directly quantified by calculating the amount of bound chloride, where higher binding capacity indicates improved resistance to chloride invasion. In the case of externally penetrated chloride, the chloride ion concentration of the immersed solution (*P*, in mol/L) was measured via automatic potentiometric titration based on the consumption of AgNO_3_ titrant:

(3) $$\;P=\frac{C_{{\mathrm{AgNO}}_{3}}\times V_{1}}{V_{2}}\;$$

where *C_AgNO_*_3_ is the concentration of the AgNO_3_ titrant (mol/L), *V*_1_ represents the volume of AgNO_3_ consumed during titration, and *V*_2_ represents the volume of the supernatant solution extracted for measurement. The chloride binding capacity was expressed as the amount of bound chloride per gram of cement, calculated using Equation (4):

(4) $$C_{b}=\frac{(P_{0}-P)\cdot V_{N}\cdot M_{\mathrm{Cl}}}{m_{p}}$$

where *C_b_* is the chloride binding capacity (mg/g)*, P*_0_ and *P* are the chloride ion concentrations (mol/L) of the solution before and after immersion, *V_N_* is the volume of the NaCl solution used (mL), *m_p_* is the immersed mass of the cement sample (g), and $M_{\mathrm{Cl}}$ is the molar mass of Cl^−^ (35.5 g/mol).

Under the condition of internal admixture, the chloride binding ratio (denoted as *β*) represents the proportion of chloride ions captured within the cement. The total chloride content *C_t_* in the sample is determined by the amount of NaCl added during specimen preparation, while the free chloride content was determined by potentiometric titration. The total chloride content and free chloride content are calculated according to Equation (5):

(5) $$C_{t}=\frac{N\cdot M_{\mathrm{Cl}}}{m_{t}},\;C_{f}=\frac{P\cdot V_{w}\cdot M_{\mathrm{Cl}}}{m_{p}}$$

where *C_t_* and *C_f_* are the total and free chloride content in the sample, *N* represents the concentration of NaCl added during sample preparation, *m_t_* indicates the total mass of cement mortar during sample preparation, and *V_w_* represents the volume of deionized water that immersed the cement powder. The chloride binding ratio β  is calculated according to Equation (6):

(6) $$\beta =\frac{C_{t}-C_{f}}{C_{t}}\times 100\%\;\;$$

(4) X-ray Diffraction and Thermogravimetric Analyses

Upon reaching the designated curing ages, hydration of the specimens was terminated by immersion in anhydrous ethanol, followed by a 24 h oven-drying at 60 °C. The dried samples were then ground to a powder with a particle size ≤0.075 mm and stored in sealed containers prior to testing. XRD analysis was conducted to qualitatively identify the hydration producst present in each sample. The test conditions were 40 kV, 40 mA with Cu Kα radiation, a scanning speed of 5°/min, a step size of 0.02°, and a 2θ range of 5–90°. Thermogravimetric (TG) analysis was performed to quantitatively characterize the hydration products. The specimens were heated from 30 °C to 900 °C at a rate of 10 °C/min under a nitrogen atmosphere.

## 3. Experimental Results and Analysis

### 3.1. Hydration Products of Anhydrous Calcium Sulfoaluminate

**Figure 2:**
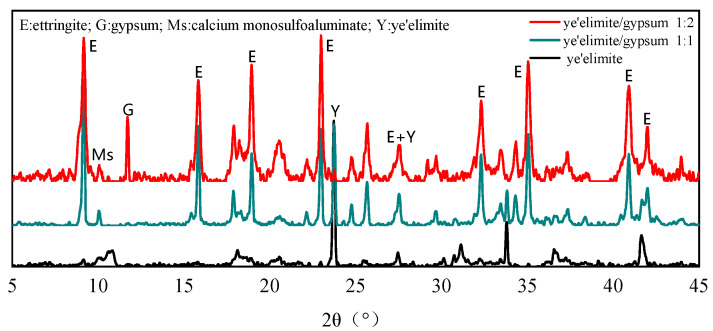
XRD patterns of $C_{4}A_{3}\overset{-}{S}$ hydration products under varying mix proportions.

Figure 2 shows the phase composition of hydration products in $C_{4}A_{3}\overset{-}{S}$ paste with different gypsum dosages after 28 days of curing. It can be concluded that the molar ratio of $C_{4}A_{3}\overset{-}{S}$-to-gypsum determines the relative amounts of AFm and AFt phases. When no gypsum is added, obvious AFm diffraction peaks can be observed, while the diffraction peaks of AFt are very weak, which indicates that the hydration of pure $C_{4}A_{3}\overset{-}{S}$ mainly generates the AFm phase as the dominant product alongside a small amount of AFt. At a $C_{4}A_{3}\overset{-}{S}$-to-gypsum molar ratio of 1:1, AFt becomes the primary hydration product, and AFm is almost undetectable due to the limited formation. Meanwhile, no diffraction peaks of gypsum are detected, suggesting that it is completely consumed during hydration. When the $C_{4}A_{3}\overset{-}{S}$-to-gypsum ratio increases to 1:2, the AFt diffraction peaks are further intensified, while a certain amount of unreacted gypsum remains in the product after the hydration reaction due to incomplete hydration [26]. In this case, the content of the AFm phase is further reduced. Moreover, the diffraction peak intensity of $C_{4}A_{3}\overset{-}{S}$ (ye’elimite) is greatly decreased, which indicates that the incorporation of gypsum accelerates the hydration of $C_{4}A_{3}\overset{-}{S}$ and plays a critical role in controlling the AFm/AFt balance. An increased gypsum dosage promotes greater AFt formation, which in turn affects the chloride binding capacity of the system.

### 3.2. Chloride Binding of Externally Penetrated Chloride Ions

(1) Chloride Binding Capacity

**Figure 3:**
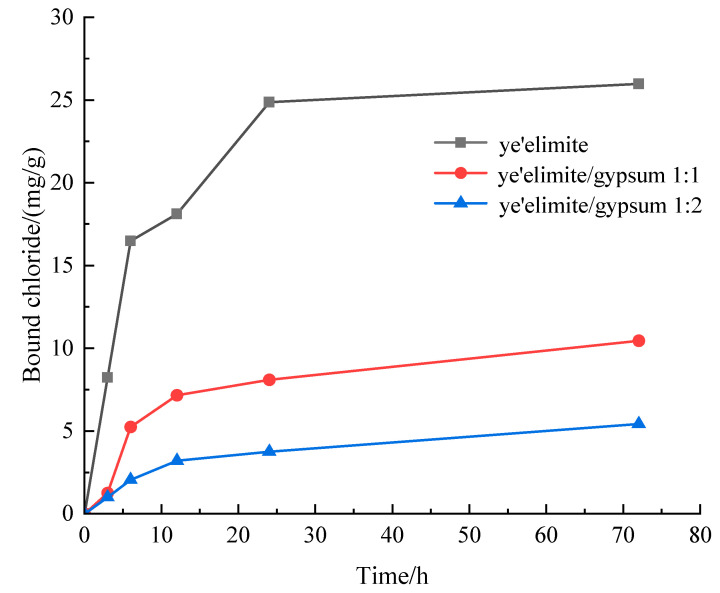
Evolution of bound chloride content of SAC samples with different immersion durations.

Figure 3 presents the chloride binding capacity of specimens with different mixing proportions. The chloride binding capacity of all three specimen groups increases with prolonged immersion. The specimen without gypsum (G-0) exhibits the highest binding capacity, whereas the specimen with the highest gypsum content (G-2) shows the lowest. This difference is attributed to the fact that the hydration products of pure $C_{4}A_{3}\overset{-}{S}$ primarily comprise AFm and AH_3_. Chloride ions can undergo ion exchange with SO_4_^2−^ in AFm to generate Friedel’s salt, and extended immersion facilitates more complete Cl^−^ binding by AFm [27]. Notably, Cl^−^ binding increases rapidly within the initial 10 h of immersion, followed by a gradual slowdown until reaching equilibrium. This is because the powder sample used for immersion has a larger contact area with the immersion liquid, which accelerates the binding of chloride ions by the hydration product. With increasing gypsum dosage, the AFm content in the hydration products decreases. At the highest gypsum level, AFt and AH_3_ become the primary hydration products, and AFt exhibits negligible chloride binding capacity; in this case, the limited binding is likely due to physical adsorption by AH_3_.

**Figure 4:**
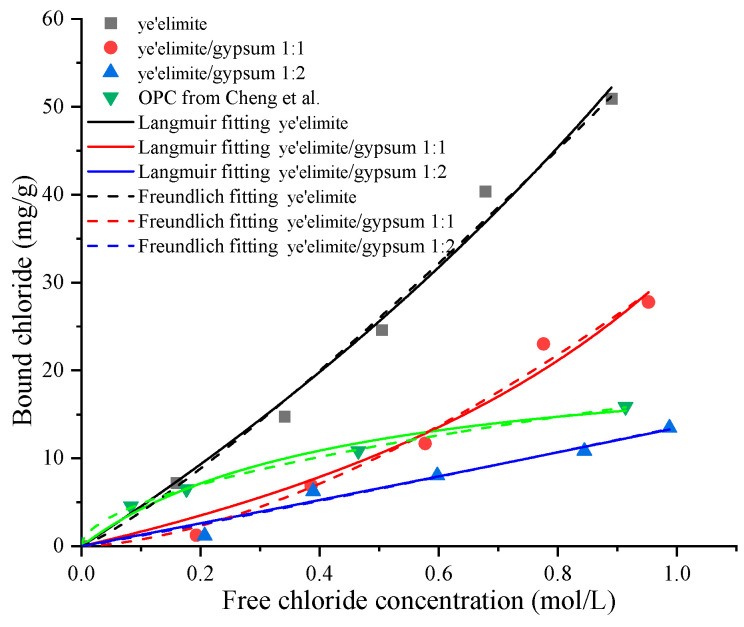
Chloride binding isotherms of specimens with different mixing proportions and OPC data from Cheng et al. [19].

Figure 4 illustrates the chloride binding isotherms of specimens with different amounts of gypsum. It can be found that the chloride concentration in the externally penetrated Cl^−^ environment also influences the chloride binding capacity of $C_{4}A_{3}\overset{-}{S}$, which increases with rising Cl^−^ concentration in the immersion solution. This is consistent with observations in Portland cement systems [28]. Elevated Cl^−^ concentrations enhance both physical and chemical binding mechanisms [29]. Similar to the time-dependent chloride binding experiment, the bound chloride content reduces with the increase of gypsum dosage. Among the three groups, G-0 exhibits the most pronounced increase in binding capacity, whereas G-1 and G-2 show more moderate increases, indicating that gypsum content significantly influences the chloride binding properties of $C_{4}A_{3}\overset{-}{S}$. Although higher Cl^−^ concentrations in the pore solution correspond to greater binding capacity, a saturation threshold exists for a given quantity of cementitious material, beyond which binding levels off. In addition, when compared to the chloride binding capacity of OPC [19], the G-0 specimen exhibits a larger chloride binding capacity. For the G-1 specimen, when the concentration of the immersion solution is lower than 0.6 mol/L, OPC shows a stronger binding capacity; however, when the concentration of the immersion solution is higher, the binding capacity of the G-1 sample surpasses that of OPC. In contrast, the binding capacity of the G-2 specimen is consistently lower than that of OPC. Previous studies [19,20] also found that commercial SAC exhibits lower chloride binding capacity than that of OPC. This indicates that the ye’elimite/gypsum ratio in commercial SAC may be between 1:1 and 1:2. According to chloride binding isotherms, the chloride binding capacity of SAC can be enhanced by adjusting the ratio of clinker to gypsum.

Furthermore, the relationship between the free chloride ion concentration and the corresponding bound chloride content can be described using equilibrium isotherms. In addition, the Freundlich and Langmuir isotherms are two widely used models, expressed as:

(7) $$\mathrm{Langmuir}\;\mathrm{isotherm}:\;C_{b}=\frac{\alpha C_{f}}{1+\beta C_{f}}$$

(8) $$\mathrm{Freundlich}\;\mathrm{isotherm}:\;C_{b}=\alpha C_{f}^{\beta }$$

where *C_f_* and *C_b_* represent the free chloride ion concentration and bound chloride content, respectively, while α and β are the chloride binding coefficients. The Langmuir and Freundlich isotherm curves for the three specimen groups, obtained through nonlinear regression analysis, are also plotted in Figure 4, and the corresponding binding coefficients together with correlation coefficients are summarized in Table 3. The *C_b_-C_f_* relationships for all specimens align well with both isotherm models, which enable us to predict and quantify chloride binding performance under different chloride exposure conditions.

(2) Characterization of hydration products after chloride binding

**Figure 5:**
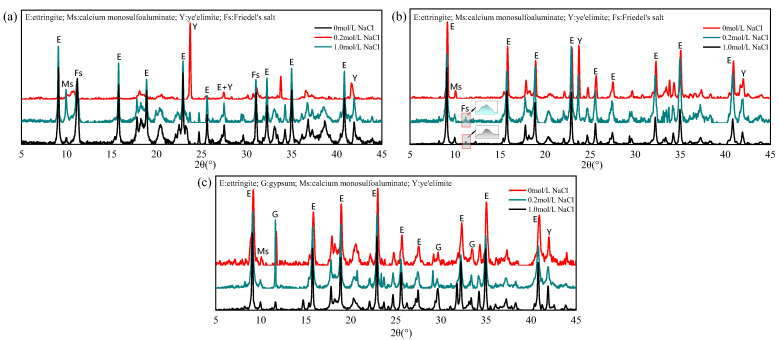
XRD patterns of different mixing proportions: (**a**) $C_{4}A_{3}\overset{-}{S}$ (G-0); (**b**) $C_{4}A_{3}\overset{-}{S}$/gypsum 1:1 (G-1); (**c**) $C_{4}A_{3}\overset{-}{S}$/gypsum 1:2 (G-2).

Figure 5 presents the XRD patterns of hydration products in $C_{4}A_{3}\overset{-}{S}$-based specimens before and after immersion in 0.2 mol/L and 1.0 mol/L NaCl solutions. For the G-0 specimen, obvious Friedel’s salt diffraction peaks appeared after immersion, confirming the reaction between hydration products and penetrated chloride ions. As the chloride concentration increased from 0.2 mol/L to 1.0 mol/L, the intensity of the AFm diffraction peak significantly decreased while the diffraction peak of Friedel’s salt increased. This indicates that as the chloride ion concentration in the immersion solution increases, more AFm chemically binds chloride ions and converts into Friedel salts. This result is consistent with the earlier conclusion that higher external chloride concentrations enhance the chloride binding capacity. The AFm phase is defined as a calcium-aluminum layered double hydroxide and consists of a positively charged main layer [Ca_2_Al(OH)_6_]^+^ and a negatively charged intermediate layer X^−^, such as a singly charged anion or a half double charged anion. In the hydration system of SAC, the interlayer anions are mainly SO_4_^2−^ and undergo competitive exchange with Cl^−^, and Cl^−^ replaces SO_4_^2−^ to form Friedel’s salt. Notably, a clear AFt peak was observed, and the peak of unhydrated ye’elimite was significantly reduced in the G-0 specimen, which was absent prior to immersion. This phenomenon can be attributed to chloride-induced sulfate displacement in AFm phases, whereby the released SO_4_^2−^ ions increase the sulfate concentration in the pore solution and subsequently promote AFt formation through secondary hydration of a large amount of unhydrated $C_{4}A_{3}\overset{-}{S}$ (ye’elimite) [22,30].

In the G-1 specimen, only weak Friedel’s salt peaks were detected, which is consistent with its limited AFm content and correspondingly reduced chloride binding capacity. As for the G-2 specimen, strong unreacted gypsum diffraction peaks along with residual AFm signals can be observed, but Friedel’s salt is basically invisible. This behavior can be attributed to the elevated SO_4_^2−^ concentrations resulting from excess gypsum, which inhibit chloride-sulfate ion exchange in AFm phases and thereby suppress chemical chloride binding. Previous studies have demonstrated that AFt phases possess negligible chloride binding capacity, with chloride uptake mainly governed by chemical binding in AFm and physical adsorption by AH_3_ [31]. Shakouri et al. [20] suggested that SAC lacks hydration products capable of chemically binding chloride ions, with AH_3_ considered the primary phase responsible for chloride binding, which is the main reason for the poor chloride binding capacity of SAC. However, the binding results indicate that $C_{4}A_{3}\overset{-}{S}$ also possesses a certain capacity for the chemical binding of chloride ions, but the chemical chloride binding may be inhibited due to the high gypsum content in commercial cement.

(3) TG analysis

**Figure 6:**
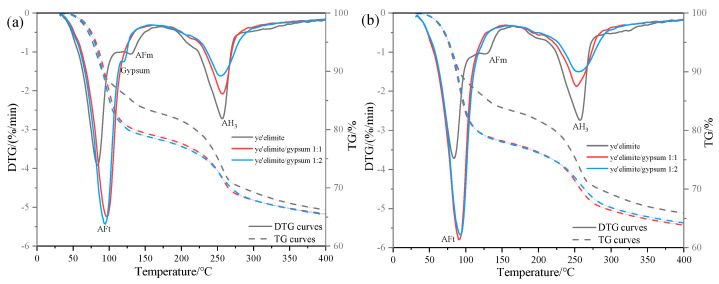
TG results under external chloride exposure conditions with varying solution concentrations: (**a**) 0.2 mol/L Cl^−^ solution, (**b**) 1.0 mol/L Cl^−^ solution.

Figure 6 illustrates the thermogravimetric (TG) profiles of three specimen groups immersed in 0.2 mol/L and 1.0 mol/L NaCl solutions. In the anhydrous calcium sulphoaluminate hydration system, the first weight loss peak observed between 30 and 120 °C corresponds to AFt dehydration, which is consistent with XRD identification of AFt formation in the G-0 specimen. Notably, AFt content in G-1 and G-2 specimens remained essentially the same at both NaCl concentrations, suggesting that AFt plays only a minor role in chloride ion binding. The subsequent weight loss peak between 120 and 150 °C is attributed to AFm dehydration. In gypsum-doped specimens (G-1 and G-2), the limited AFm formation resulted in a weakening of the dehydration peak, directly accounting for their lower chloride binding capacity. Another obvious weight loss peak occurs between 180 and 280 °C, which is associated with the release of bound water in AH_3_, and this weight loss peak partially overlaps with the dehydration range of 210–360 °C of Friedel’s salt [28]. Therefore, the gradual decrease in the intensity of the weight loss peak in this range with increasing gypsum content indicates that the amount of Friedel salt formation is also decreasing, which is consistent with the XRD results and the total chloride binding measurements. The gypsum-modified system mainly relies on physical adsorption dominated by AH_3_ to achieve Cl^−^ binding—a mechanism that is less stable and less effective than AFm-driven chemical binding. This explains the lower chloride binding efficiency observed in gypsum-doped specimens.

Comparative analysis of TG results under varying Cl^−^ concentrations reveals contrasting behaviors. In gypsum-free systems, the increase in Cl^−^ content was associated with AFm depletion and enhanced Friedel’s salt formation, demonstrating a dominant role of AFm in chemical chloride binding. By contrast, gypsum-containing systems exhibited stable AFt content regardless of Cl^−^ concentration, since the presence of sulfate ions (SO_4_^2−^) causes $C_{4}A_{3}\overset{-}{S}$ hydration to mainly form the Aft rather than the AFm phase. The high crystallinity and needle-like morphology of AFt densify the hardened paste matrix, effectively inhibiting Cl^−^ ingress, and the chemical binding of Cl^−^ is achieved by the ion exchange between Cl^−^ and SO_4_^2−^ in the interlayer of AFm. In addition, when the concentration of SO_4_^2−^ in the system is too high (in the case of the $C_{4}A_{3}\overset{-}{S}$/SO_4_^2−^ ratio being 1:2), it may prevent the SO_4_^2−^ in the interlayer of AFm from being displaced, which in turn impairs the chloride ion binding capacity of the system. As a result, the chloride binding capacity of gypsum-modified systems showed only a modest increase with rising solution concentration, explaining the relatively gentle upward trend in binding efficiency observed with gypsum incorporation.

### 3.3. Chloride Binding of Internally Incorporated Chloride Ions

(1) Chloride binding capacity

**Figure 7:**
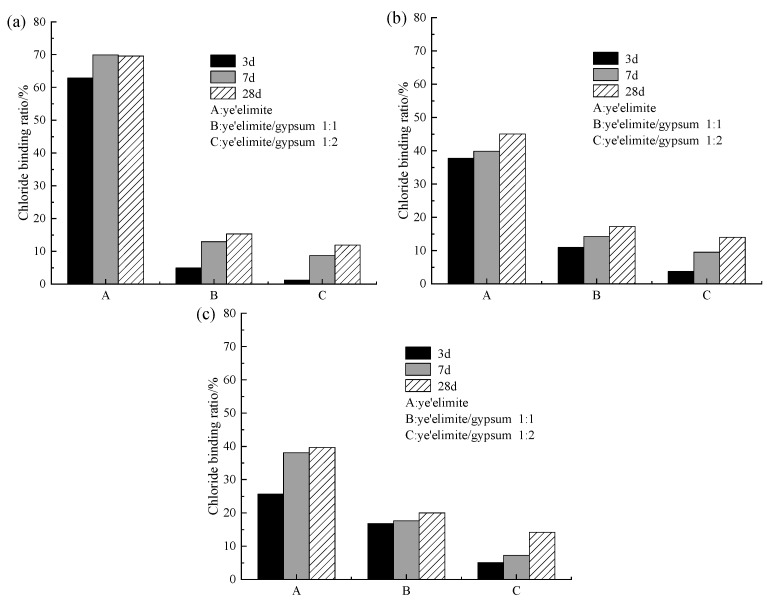
Chloride binding capacity under internally incorporated chloride conditions: (**a**) 0.02 mol; (**b**) 0.04 mol; (**c**) 0.06 mol.

Figure 7 presents the chloride binding ratios under different levels of incorporated Cl^−^. As shown in Figure 7, both curing age and Cl^−^ dosage are the dominant factors influencing chloride binding under internal incorporation conditions. Across all three dosage groups, a consistent trend is observed: the chloride binding ratio generally increases with increasing curing age. This is consistent with the results obtained by Wang et al. [17] and Zhao et al. [32] that the CBR of FAC to premixed chloride increases with curing time. Extended curing age facilitates the generation of additional hydration products that strengthen chloride binding. These contributions arise from the physical adsorption by AH_3_, the chemical exchange between AFm phases and Cl^−^ leading to Friedel’s salt formation, and the direct participation of chloride ions in cement hydration. Together, these mechanisms lead to the enhancement of the overall chloride binding capacity of cement paste with curing age.

As for the variation in chloride binding ratio under different Cl^−^ dosages, specimen G-0 shows a decreasing Cl^−^ binding ratio with increasing incorporated Cl^−^ content. Since pure $C_{4}A_{3}\overset{-}{S}$ hydrate samples exhibit a high chloride binding capacity, the proportion of bound chloride content is relatively high at low chloride ion dosages. However, as the chloride dosage increases, although the absolute amount of bound chloride also rises, the increment is limited. Consequently, the ratio of bound chloride to total chloride gradually decreases with increasing chloride dosage. By contrast, specimens G-1 and G-2 show lower sensitivity to changes in Cl^−^ dosage, which may be attributed to the finite maximum chloride binding capacity inherent to the cementitious matrix. As reported in previous studies [33], the coexistence of SO_4_^2−^ and Cl^−^ preferentially promotes AFt formation. However, AFt provides much weaker chloride binding compared with Friedel’s salt, and the crystallization of AFt also inhibits the nucleation of Friedel salt.

(2) Characterization of hydration products post chloride binding

**Figure 8:**
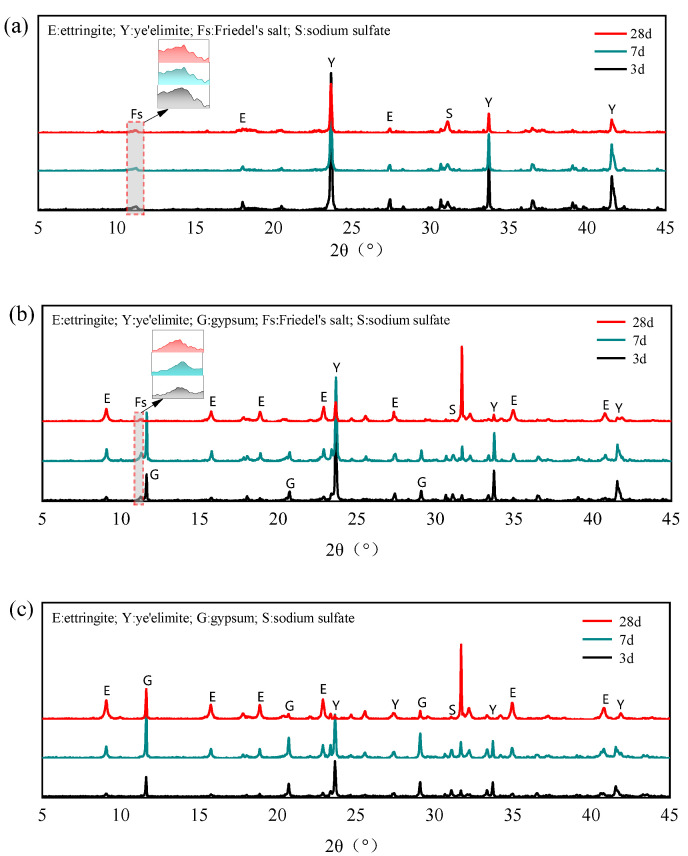
XRD patterns of different mixing proportions with 0.4 mol Cl^−^: (**a**) $C_{4}A_{3}\overset{-}{S}$; (**b**) $C_{4}A_{3}\overset{-}{S}$/gypsum 1:1; (**c**) $C_{4}A_{3}\overset{-}{S}$/gypsum 1:2.

The XRD patterns of different mixing proportions with 0.4 mol NaCl under different curing ages are shown in Figure 8. After adding NaCl to the hydration system of $C_{4}A_{3}\overset{-}{S}$, no obvious AFm diffraction peaks can be observed even in the G-0 specimen, whereas distinct Friedel’s salt diffraction peaks appeared. Berger et al. also observed similar results that when chloride ions were present in the hydration environment of SAC, Friedel’s salts were rapidly formed instead of AFt and AFm phases [34]. In addition, the diffraction peaks of Na_2_SO_4_ are detected in the G-0 specimen, which further indicates that the internally incorporated Cl^−^ participates in the hydration of anhydrous calcium sulphoaluminate, directly generating both Friedel’s salt and Na_2_SO_4_ through the reaction mechanism outlined in Equation (9). In addition, the AFm phases generated by normal hydration in cement can further react with Cl^−^ to generate Friedel’s salt, leading to the gradual depletion of AFm phases in the hydration products.

(9) $$C_{4}A_{3}\overset{-}{S}+2\mathrm{NaCl}+16H\to C_{3}A\cdot {\mathrm{CaCl}}_{2}\cdot 10H+{\mathrm{Na}}_{2}{\mathrm{SO}}_{4}+2AH_{3}$$

For specimens G-1 and G-2, trace amounts of Friedel’s salt formed during hydration with NaCl. Wei et al. [35] also found that only a small amount of Friedel’s salt is generated in SAC with Cl^−^ incorporation. They suggested that an excess of SO_4_^2−^ is unfavorable for the formation of Friedel’s salt and attributed the binding ability of SAC to the C-S-H. By isolating $C_{4}A_{3}\overset{-}{S}\;$ and investigating the hydration products, the formation of Na_2_SO_4_ can be found, which demonstrates that Cl^−^ can still participate in the hydration of anhydrous calcium sulphoaluminate to generate Friedel’s salt even in the presence of gypsum. In addition, Cl^−^ acts as a strong electrolyte, and when incorporated into cement paste, it significantly accelerates the early-stage hydration of anhydrous calcium sulphoaluminate [36], thereby promoting the rapid formation of AFt and enhancing its crystallinity. This preferential hydration pathway restricts the extent of chloride binding through Friedel’s salt formation, ultimately resulting in lower overall chloride binding capacity in gypsum-containing specimens compared to the G-0 specimen.

(3) TG analysis

**Figure 9:**
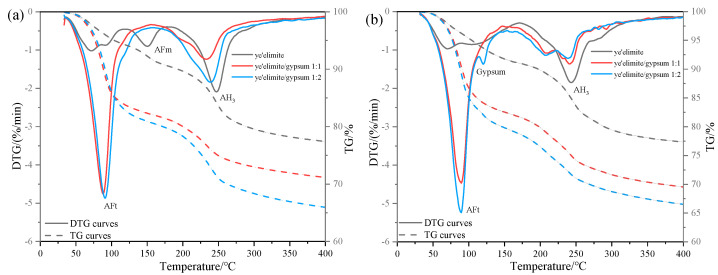
TG results under internal chloride incorporation conditions with varying compositions: (**a**) non-chlorinated control; (**b**) 0.04 mol chloride incorporated system.

Figure 9 compares the TG results of specimens before and after internal NaCl incorporation after 28 days of hydration. As shown, the original AFm mass loss peak disappears in the G-0 specimen after Cl^−^ incorporation, indicating that the presence of Cl^−^ leads to a reduction or even elimination of AFm phases in the hydration products. This finding is consistent with the earlier XRD results, which confirmed the absence of AFm diffraction peaks and the emergence of Friedel’s salt. For specimens G-1 and G-2, no distinct AFm mass loss peaks were detected either before or after Cl^−^ incorporation. Combined with the previously demonstrated influence of gypsum dosage on chloride binding capacity under internal Cl^−^ incorporation conditions, this observation confirms that the superior chloride binding capacity of specimen G-0 arises not only from the direct reaction between $C_{4}A_{3}\overset{-}{S}$ and Cl^−^ to form Friedel’s salt but also from the substantial AFm phase formation during $C_{4}A_{3}\overset{-}{S}$ hydration, which significantly augments Cl^−^ binding via an ion-exchange reaction.

In contrast, the hydration products of G-1 and G-2 specimens are primarily composed of AFt and AH_3_. Comparative TG analyses of systems with and without internal Cl^−^ incorporation show nearly identical mass-loss patterns associated with these phases. This indicates that internally incorporated Cl^−^ has little influence on the formation or stability of AFt and AH_3_. Together with the pronounced reduction in chloride binding capacity observed in gypsum-containing systems, these results demonstrate that AFt contributes negligibly to chloride binding, while the physical adsorption capacity of AH_3_ serves as the dominant mechanism of Cl^−^ sequestration in these formulations.

**Figure 10:**
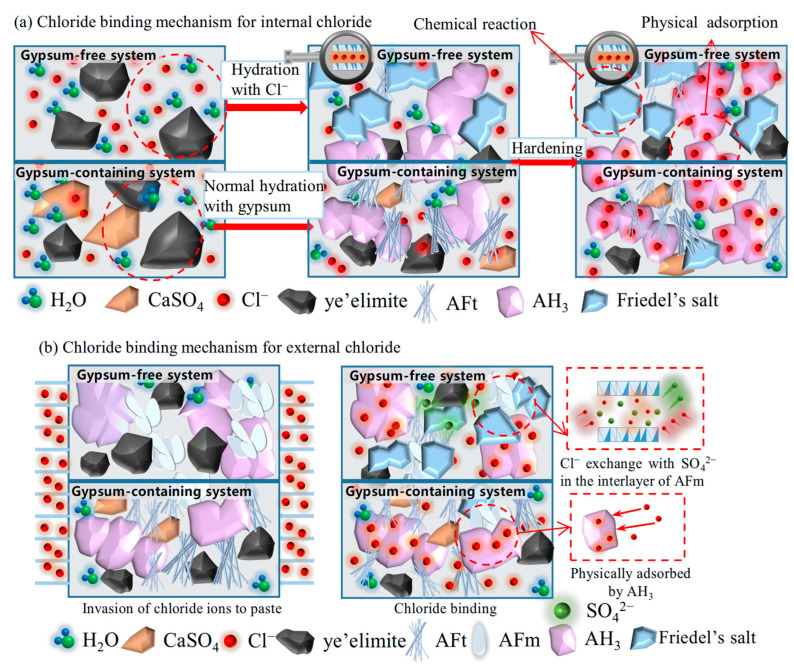
Chloride binding mechanism of $C_{4}A_{3}\overset{-}{S}$ for internal and external chlorides.

Based on the chloride binding results obtained from experiments, the chloride binding mechanism of $C_{4}A_{3}\overset{-}{S}$ for internal and external chlorides is illustrated in Figure 10. Internal chloride ions are directly added into the mixing water; in a gypsum-free system, the internally incorporated Cl^−^ participates in the hydration of $C_{4}A_{3}\overset{-}{S}$, directly generating Friedel’s salt and AH_3_, thereby being chemically bound. The formed AH_3_ will further physically adsorb the residual free Cl^−^. While in a gypsum-containing system, $C_{4}A_{3}\overset{-}{S}$ will react preferentially with gypsum to form AFt, which has almost no binding ability; only a small proportion of Cl^−^ also reacts with $C_{4}A_{3}\overset{-}{S}$ to form Friedel’s salt. In addition, Cl^−^ can still be physically adsorbed by generated AH_3_. External chloride ions penetrate from immersion solution to hydrated $C_{4}A_{3}\overset{-}{S}$ pastes. The main compositions for the gypsum-free system are AFm, AH_3_, and unhydrated $C_{4}A_{3}\overset{-}{S}$, Cl^−^, which will exchange SO_4_^2−^ in the interlayer of AFm to form Friedel’s salt and are chemically bound through interaction with AFm. In a gypsum-containing system, a limited amount of AFm is generated, and the high concentration of SO_4_^2−^ inhibits chloride-sulfate ion exchange in AFm phases. Similar to the internal incorporation condition, penetrated Cl^−^ can also be adsorbed by the AH_3_ gels for physical binding.

## 4. Conclusions

This study investigates the chloride binding mechanisms of anhydrous calcium sulphoaluminate under external Cl^−^ penetration and internal Cl^−^ incorporation conditions. A series of experiments with varying gypsum dosages, supported by XRD and TG analyses, led to the following conclusions:

1. The phase composition of hydration products is strongly influenced by gypsum dosage. In gypsum-rich systems, AFt is the main hydration product, whereas in low-gypsum systems, AFm formation is significantly enhanced. The AFm/AFt ratio is a critical factor governing chloride binding capacity.
2. Under external chloride exposure, binding occurs through the combined contributions of AFm and AH_3_. AFm binds chloride via SO_4_^2−^/Cl^−^ ion exchange to form Friedel’s salt, while AH_3_ contributes through physical adsorption. For internal chloride, binding arises not only from the chemical reaction of AFm and the physical adsorption of AH_3_, but also from the direct reaction between anhydrous calcium sulphoaluminate and incorporated chloride, leading to Friedel’s salt formation.
3. In external chloride environments, binding capacity increases with solution concentration and exposure duration until reaching a dynamic equilibrium, controlled by the saturation of AFm ion-exchange sites and the adsorption limit of AH_3_. For internal chloride, prolonged curing promotes AFm formation and thereby enhances binding capacity. Increasing chloride dosage slightly reduces binding in gypsum-free systems, while gypsum-containing systems exhibit limited sensitivity to dosage variations.
4. Gypsum-free formulations demonstrate superior chloride binding performance due to abundant AFm formation. In contrast, gypsum incorporation favors AFt formation, which has intrinsically lower binding capacity, leading to reduced overall performance.

The investigation of the chloride binding behavior and mechanism of anhydrous calcium sulphoaluminate can help to provide valuable guidance for the design of SAC-based concrete structures in marine environments. Based on the observed influence of gypsum on the chloride binding capacity, the gypsum dosage can be optimized to prevent the inhibition of Cl^−^ binding, thereby reducing the risk of steel corrosion and extending the service life of reinforced structures. It should also be noted that other factors—such as pore solution chemistry, pH, and temperature—may exert significant effects on chloride binding capacity. These aspects require further investigation through both theoretical analysis and experimental validation.

## Figures and Tables

**Table 1:** Chemical compositions of the prepared $C_{4}A_{3}\overset{-}{S}$.

Compositions (%)	SiO_2_	Al_2_O_3_	CaO	SO_3_	P_2_O_5_	Na_2_O	Fe_2_O_3_	SrO
$C_{4}A_{3}\overset{-}{S}$	0.19	49.52	36.62	13.35	0.03	0.16	0.06	0.07

**Table 2:** Experimental mix proportion design.

Sample Number	W/C	Water (g)	$C_{4}A_{3}\overset{-}{S}$ (g)	Gypsum (g)
G-0	0.4	20	50	0
G-1	0.4	20	39	11
G-2	0.4	20	32	18

**Table 3:** Fitting parameters of Langmuir and Freundlich isotherms for the three specimen groups.

Sample	Langmuir Isotherm			Freundlich Isotherm		
	*α*	*β*	R^2^	*α*	*β*	R^2^
G-0	43.99	−0.28	0.98	58.69	1.18	0.99
G-1	15.72	−0.51	0.97	31.24	1.62	0.98
G-2	12.91	−0.04	0.96	13.53	1.05	0.96

## Data Availability

The original contributions presented in this study are included in the article. Further inquiries can be directed to the corresponding author.
